# Visual automated macromolecular model building

**DOI:** 10.1107/S0907444913000565

**Published:** 2013-03-14

**Authors:** Gerrit G. Langer, Saul Hazledine, Tim Wiegels, Ciaran Carolan, Victor S. Lamzin

**Affiliations:** a European Molecular Biology Laboratory, c/o DESY, Notkestrasse 85, 22603 Hamburg, Germany

**Keywords:** model building, *ARP*/*wARP*, molecular graphics

## Abstract

Automated model-building software aims at the objective interpretation of crystallographic diffraction data by means of the construction or completion of macromolecular models. Automated methods have rapidly gained in popularity as they are easy to use and generate reproducible and consistent results. However, the process of model building has become increasingly hidden and the user is often left to decide on how to proceed further with little feedback on what has preceded the output of the built model. Here, *ArpNavigator*, a molecular viewer tightly integrated into the *ARP*/*wARP* automated model-building package, is presented that directly controls model building and displays the evolving output in real time in order to make the procedure transparent to the user.

## Abstract 2

The molecular viewer *ArpNavigator* allows easy execution of *ARP*/*wARP* model-building routines while model-update steps are shown in real time, rendering the whole process transparent to the user.

## 1. Introduction

Until the relatively recent advent of automated model-building software, macromolecular crystallographic model building was an essentially manual process involving the extensive use of three-dimensional interactive graphical editors in order to locate and manipulate individual atoms and rigid atomic groups in electron density. Such editors included the pioneering software *FRODO* (Jones, 1978 ▶) and its advanced successor *O* (Jones *et al.*, 1991 ▶). The use of more automated model-building software such as *RESOLVE* (Terwilliger *et al.*, 2008 ▶), *Buccaneer* (Cowtan, 2006 ▶) and *ARP*/*wARP* (Langer *et al.*, 2008 ▶) has greatly accelerated the various steps of structure determination and, most importantly, has the scientific benefit of generating reproducible and consistent results. This does not, of course, mean that these results are without error, but the level to which it has led to an improvement in the dissemination of incorrect models was emphasized two decades ago (Brändén & Jones, 1990 ▶). However, by removing the human component from the model-building procedure, automated software has reduced the motivation for novice users to understand how their protein model was formed from initial electron density. The evolution of a built model and the related improvement of the electron density have become less obvious. Nowadays, less experienced users do not have much information at hand on how the automatically built but possibly incomplete or partially incorrect model can be improved further and optimal modelling accomplished.

Current graphical user interfaces (GUIs) for crystallo­graphic modelling, such as *CCP*4*i* (Winn *et al.*, 2011 ▶) and the *PHENIX* GUI (Echols *et al.*, 2012 ▶), are primarily focused on receiving input parameters from a user in order to launch a computational job according to a pre-defined protocol. Some interfaces can also communicate with external third-party molecular viewers, such as *Coot* (Emsley *et al.*, 2010 ▶) or *PyMOL* (Schrödinger; http://www.pymol.org/), in order to display the results of completed model-building iterations. Some crystallographic computational tasks take milliseconds on modern computers, but many require a significant amount of time (minutes to days). When the results are displayed after a long delay, the real-time picture of the model building is no longer of practical benefit because users switch to other tasks. Moreover, using disconnected software solutions for computation and for display necessitates a working knowledge of both systems, and this can be challenging for new users.

In this manuscript, we offer our view on circumventing the problems described and present our first results implemented in the newly completed software *ArpNavigator*. This is a molecular viewer that began as an experimental addition to the *ARP*/*wARP* software suite. It has now matured to become the primary intuitive interface for interacting with the automated model-building processes in real time, and can be of use to both experienced and novice users. *ArpNavigator* is tightly integrated into *ARP*/*wARP*, having a similar software structure and using the same libraries. It controls the setup of computational tasks and displays the intermediate steps of the model-building process. In addition, *ArpNavigator* also provides complementary functionality to access *ARP*/*wARP* facilities for exploratory analysis and model completion, such as ligand identification and fitting. The most frequent applications of *ArpNavigator* are described below in more detail.

## 2. Protein model building

**Figure 1:**
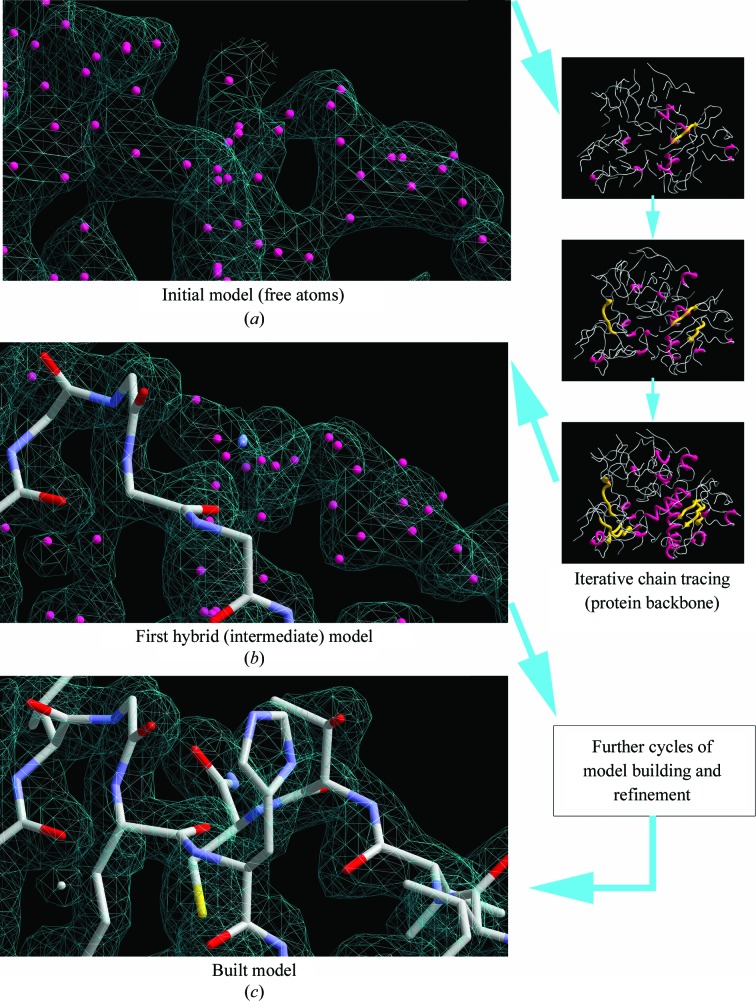
Workflow of *ArpNavigator* building a model of the 475-residue protein leishmanolysin (PDB entry 1lml; Schlagenhauf *et al.*, 1998 ▶) using 2 Å resolution data. (*a*) The free-atoms model is placed into the experimental density map and is used for iterative chain tracing to build the protein backbone, resulting in a hybrid model (*b*). The refinement and rebuilding of the hybrid model are repeated iteratively, resulting in a more complete model (*c*). All coordinate files and maps generated during this process are loaded in real time into *ArpNavigator*.

*ArpNavigator* can initiate the model-building process from either X-ray data with initial phases or from both X-ray data and an existing (molecular-replacement) model that is used as the basis for further improvement (Langer *et al.*, 2008 ▶). Similarly, as in the familiar *CCP*4*i* GUI, *ARP*/*wARP* model building is launched after the user indicates the input files and selects configuration options controlling the computational process. Once the job starts, the viewer loads the user-provided starting model or the built free-atoms model and displays the initial electron density (Fig. 1 ▶ *a*).

The protocol begins with refinement of the initial atomic model with *REFMAC* (Murshudov *et al.*, 2011 ▶) followed by several rounds of peptide-chain tracing. This leads to the first hybrid model (Fig. 1 ▶ *b*) containing both free atoms and chemically assigned atoms that make up partially built protein chains. Such hybrid models are subsequently refined, leading to better phase estimation and thus higher quality electron-density maps that are iteratively reloaded in the graphics window along with the associated atomic models. The process of protein model building and refinement continues until a predefined number of iterations has been executed, producing the final model for presentation (Fig. 1 ▶ *c*).

**Figure 2:**
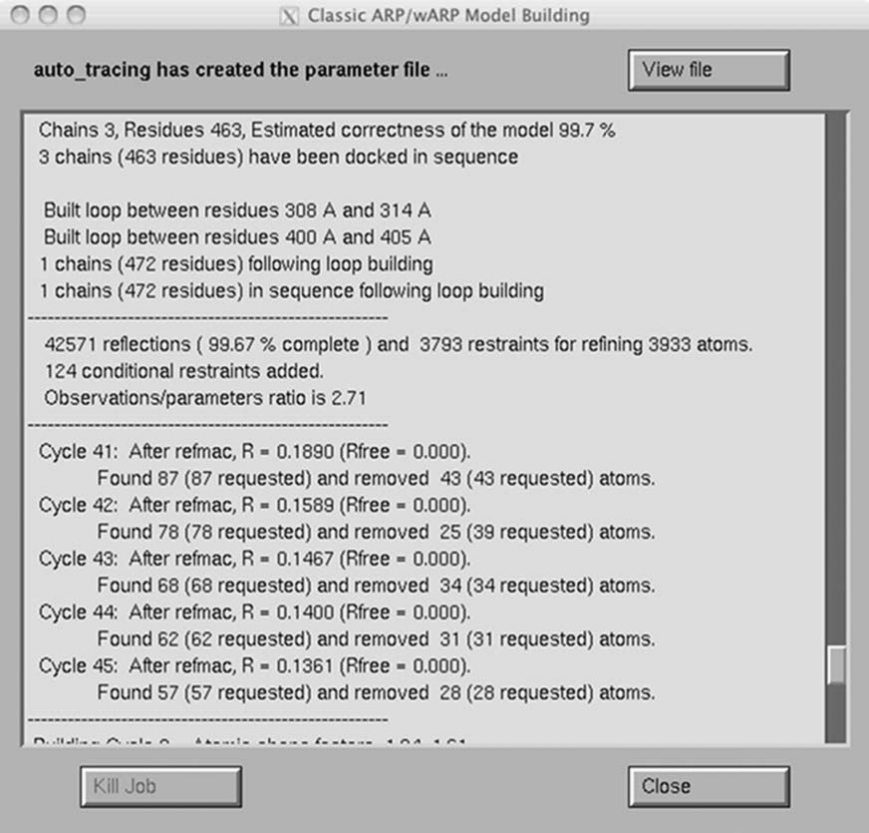
The log window that displays detailed information while building the protein structure shown in Fig. 1 ▶.

A single iteration (sometimes also called an *ARP*/*wARP* big cycle) consisting of five rounds of model building and five cycles of *REFMAC*/*ARP* refinement and update typically takes 10–20 min on a modern desktop workstation. Upon completion, the viewer has displayed 13 models and six electron-density maps. The associated visual user feedback is typically more intuitive than traditional consultation of log files, although a scrolling log is displayed in a separate window (Fig. 2 ▶), in which relevant quantitative data underlying the process are presented.

The menus, options and view of the map and protein molecules are all multi-threaded so that a user can zoom, rotate and change the display settings while model building is simultaneously taking place. Whenever a new structure is loaded, the existing orientation and zoom level is maintained.

The computational process is unperturbed by the use of *ArpNavigator* and so the resulting macromolecular structure is equivalent to that produced by *ARP*/*wARP* when controlled through the *CCP*4*i* GUI, the Unix command line or remotely over the web. The intuitive visualization of the model-building process introduced in *ArpNavigator* rather prompts the user to appropriately consider the various model-building protocols offered by *ARP*/*wARP* and to more easily choose the right approach for rapid completion of structure determination. In this regard, it is important to note that the user still has control over the most important options in *ARP*/*wARP*-based model building when it is run through *ArpNavigator*.

## 3. Model viewing

**Figure 3:**
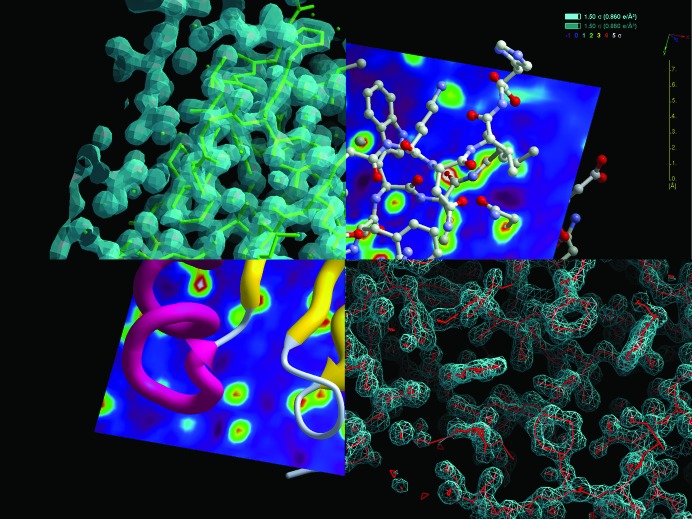
A selection of model-viewing options in *ArpNavigator* depicted for PDB structure 3l9a: the C-terminal domain of a *Streptococcus mutans* hypothetical protein (Midwest Center for Structural Genomics, unpublished work). Shown clockwise from the top left are a stick representation in solid electron density (50% opacity, 1.5σ), a ball-and-stick representation in planar density, also showing a ‘scaleometer’ and the contour levels of shown electron densities, a skeleton representation of the electron density shown as a mesh and the protein in cartoon representation in planar density.

*ArpNavigator* is able to load or compute and display electron-density maps calculated from X-ray data in the CCP4 MTZ format or saved in the CCP4 map format, as well as PDB-format coordinate files. In addition to this, reflecting the increasing use of crystallographic methods for drug discovery, the ability to load small molecules in SDF and SMILES formats has been implemented. *ArpNavigator* supports several map and structure representations. The default structural format is three-dimensional sticks with lighting effects. However, when macromolecular structures are large and their display may cause degradation of graphical performance, structures are automatically presented as lines without lighting effects. Ball-­and-stick, worms and cartoon representations can also be chosen. Electron-density maps, meanwhile, can be presented as a traditional mesh, point cloud, solid voxelated body or as a cross-section, and can be variably manipulated based on the contour level for mapping, the interpolation level or the level of clipping to a viewed structure. A selection of possible map- and model-viewing options is given in Fig. 3 ▶.

To support novice users, and as a general convenience, *ArpNavigator* automatically resizes manually loaded models and automatically controls fogging and *Z*-plane clipping while zooming in and out of models. Should this default behaviour not be to the taste of advanced users, they can control all of the above manually and, indeed, turn off fogging and lighting effects completely if desired.

## 4. Auxiliary functionality: efficient density analysis

One advantage of interactively controlling the construction of a macromolecular model from a molecular viewer is that actions can be initiated quickly by point and click rather than by filling out a web form or typing a command in a terminal window. This encourages exploratory analysis of built models or refined electron density.

**Figure 4:**
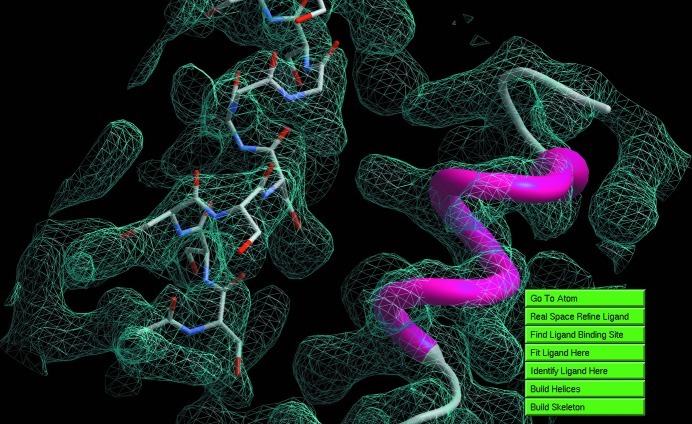
Helices built using the right-click ‘quick action’ menu. This helical substructure, computed in a few seconds on a single desktop workstation, was built into a 3.0 Å resolution electron-density map calculated from the experimental data associated with PDB entry 1c48 (345 residues; mutated shiga-like toxin; Ling *et al.*, 1998 ▶).

One example of such analysis that is available within *ArpNavigator* is a check for the interpretability of an electron-density map by rapid generation of a skeleton representation (Greer, 1974 ▶) of that density (Fig. 3 ▶, bottom right). A similarly rapid option is offered to build a protein backbone derived from secondary-structure elements, helices and strands. Both functions are accessible by right mouse clicking through a ‘quick action’ menu. Details of this helix/strand-building method will be published elsewhere. Here, we only note that the density analysis is non-iterative, and thus the results are obtained and loaded into the viewer typically within seconds (Fig. 4 ▶).

**Figure 5:**
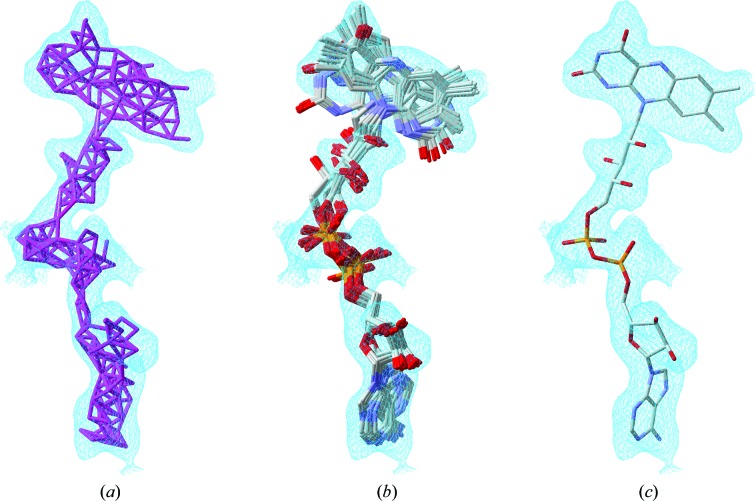
The modelling of the ligand flavin adenine dinucleotide into *p*-hydroxybenzoate hydroxylase (PDB entry 1cc6; Eppink *et al.*, 1999 ▶) as depicted by the *ArpNavigator* GUI. (*a*) Following input of the experimental 2.2 Å resolution data and the protein model, a difference density map is automatically calculated and shown along with the ‘sparse grid’ used for ligand building by the *ARP*/*wARP* ‘label-swapping’ method. (*b*) The ensemble of models built prior to selection of the best-fitting ligand model. (*c*) The final ligand model after real-space refinement is output to the screen for viewing and further analysis where necessary.

A user may wish to fit a known ligand to an electron density and, when the binding site can be specified manually, *ArpNavigator* offers such specification by a single point and click. This results in a hidden calculation of the intersection between the viewed part of the electron density and a line drawn from the viewing position. The electron-density blob containing this intersection point is then taken as the binding site for ligand placement. As for model building, the steps comprising ligand fitting are also graphically exposed in *ArpNavigator*, with all potential ligand conformations identified by the software being displayed before the final conformation is chosen and fitted (Fig. 5 ▶). Details of the algorithms used for building ligands with *ARP*/*wARP* can be found in Langer *et al.* (2012 ▶).

## 5. Implementation

*ArpNavigator* is written in C and makes use of *ARP*/*wARP* libraries written in Fortran 77/95. The three-dimensional graphics and main menus are displayed using the OpenGL fixed-function pipeline, while some dialogue boxes are displayed using X11 calls. The software remains lightweight and portable by not using a framework to control and display the graphical user interface. Multi-threading is achieved using the POSIX threads library with only one mutex in use owing to a disciplined application of atomic operations and no concurrent sharing of data structures between threads.

The molecular viewer is tightly integrated into *ARP*/*wARP* but uses the systems-design approach known as ‘loose coupling’. Communication with the rest of the *ARP*/*wARP* model-building suite takes place through Unix sockets that are connected to the top-level *ARP*/*wARP* protocols which control the dozens of individual programs that make up the model-building suite. This loose coupling has the advantage that the underlying model-building and exploratory algorithms can be added or altered without greatly impacting on *ArpNavigator*. Changes to the molecular viewer do not require modifications of the scientific algorithms and the loose coupling also ensures that the use of *ArpNavigator* does not affect the final resulting model.

## 6. Discussion

**Figure 6:**
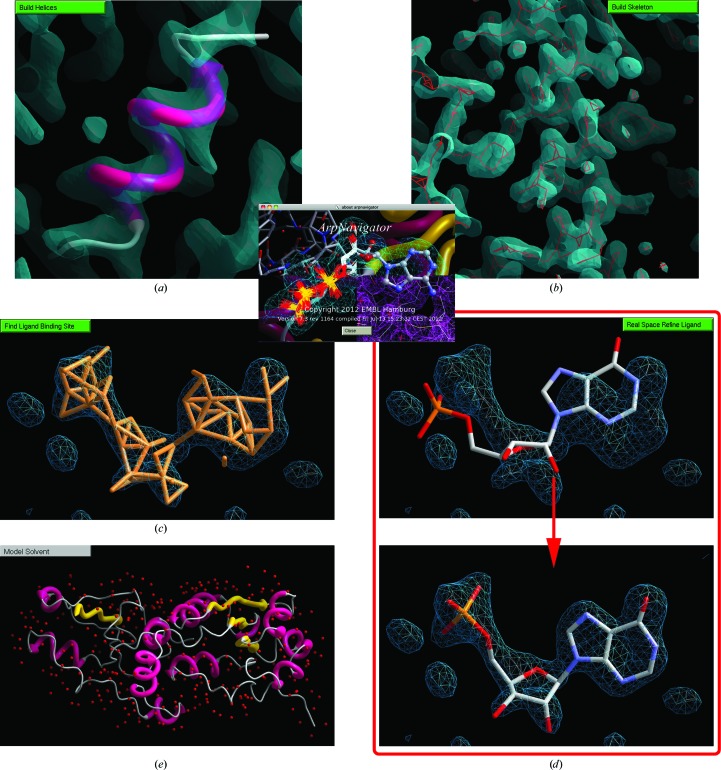
Integrative model building with *ArpNavigator*. Assessing the quality of initial electron density by building helices (*a*) or skeletons (*b*). Following model building, in the case of bound ligands the binding site can be identified (*c*) and the coarsely placed ligand refined into the map (*d*). Solvent molecules can be modelled (*e*). Contour levels are at 1.5σ in (*a*) and (*b*) and at 3.5σ in (*c*) and (*d*). The resolution of the maps used is 3.2 Å in (*a*) and 1.9 Å in (*b*), (*c*) and (*d*); all maps shown are 2*mF*_o_ − *DF*_c_ maps.

*ArpNavigator* introduces a number of novel operations that could not previously be achieved using the *CCP*4*i* interface or command line. This permits more convenient model building into an initial electron-density map (with or without some already available model), including modelling of ligands and solvent. A quick quality assessment of the initial map is possible by building helices (Fig. 6 ▶ *a*) or a skeleton (Fig. 6 ▶ *b*). Following protein model building (Fig. 1 ▶), the binding site of possible ligands can be located automatically (Fig. 6 ▶ *c*). Subsequently, a ligand can be coarsely fitted and real-space refined into the density by mouse clicking (Fig. 6 ▶ *d*). Finally, solvent can be modelled with immediate feedback to the user (Fig. 6 ▶ *e*).

**Figure 7:**
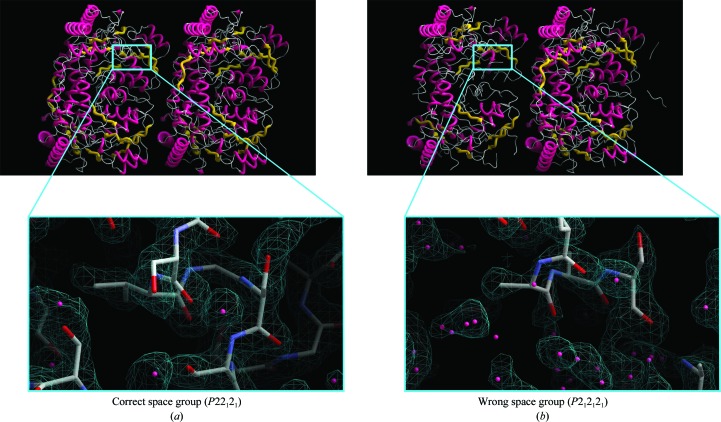
Comparison of 2*mF*_o_ − *DF*_c_ electron-density maps contoured at 1.5σ above the mean and models of an alanine-glyoxylate aminotransferase (AGT; Fodor *et al.*, 2012 ▶) built at 2.0 Å resolution (PDB entry 3r9a) in the correct space group (*a*) and the wrong space group (*b*).

Both experienced and novice structural biologists can benefit from using automated macromolecular model building and appreciate three-dimensional molecular-graphics solutions for model viewing, analysis and presentation. However, it is important that the users are given more options to understand the process that leads to an incomplete or partly incorrect structure if they are to improve and finalize the solution. In the example shown in Fig. 7 ▶, the outcome of model building following phasing by molecular replacement in two different space groups is shown. According to the standard quality indicators the choice of the correct space group during molecular replacement was not obvious, but inspection of the *ARP*/*wARP*-built model and its graphical viewing with *ArpNavigator* was decisive. Closer investigation of the electron-density maps shows that the density obtained in the wrong space group is quite discontinuous and poorly defined. More specifically, the expected number of residues for this dimeric structure was 1548. In the wrong space group (*P*2_1_2_1_2_1_) the built model comprised 1396 residues in 33 fragments and gave an *R* factor of 30%. In this model, the subunit on the right (Fig. 7 ▶ *b*) is built in eight fragments, while that on the left is built in 25 fragments. In the correct space group (*P*22_1_2_1_) the built model comprised 1519 residues in only eight fragments (four fragments per NCS-related copy) with an *R* factor of 26%, resulting in a much better electron-density map (Fig. 7 ▶ *a*). Since the structure contained NCS, the model building has additionally benefitted from the automatic detection of NCS recently incorporated into *ARP*/*wARP* (Wiegels & Lamzin, 2012 ▶).

At the same time, it is certainly true that (novice) users are more productive with software that requires less expertise to operate. In macromolecular crystallography, we are still far from somewhat further developed disciplines, such as smartphone technologies, which may not require the user to have any knowledge of the underlying principles of their functionalities. Therefore, the development of *ArpNavigator* is an attempt to balance various requirements including the tight integration with the *ARP*/*wARP* automated software suite so that the user of the viewing software interacts with the intermediate steps of the procedure while the structure is being determined.

Potential future developments of *ArpNavigator* include technical enhancements such as drag-and-drop file handling and further simplified user dialogues. Different controls for manipulating the objects in view can be made available for selection and/or modification by the user if desired. Point-and-click selection of atoms, residues or other elements of loaded models in order to aid graphical presentation may well be added. There is also an opportunity to add validation of partly built models and to point to the sections of models that may require further automatic treatment or attention from the user. Automatic completion of macromolecular models, comprising initial assessment of potential ligand-binding sites, identification of the appropriate ligands and ligand building at each such site, bookended by solvent modelling, would be a major leap that is within reach thanks to the interactivity afforded by the *ArpNavigator* software. As new functionality is added to the *ARP*/*wARP* suite, we expect that the various *ArpNavigator* interfaces will reflect these advancements.

To our knowledge, *ArpNavigator* is the first molecular viewer in macromolecular X-ray crystallography to be tightly integrated into one automated model-building package. We hope that the increased visibility of the model-building process provided by *ArpNavigator* will give the user a better understanding of crystallographic structure determination and approaches to model construction and completion.
